# Next-Generation Sequencing Analysis for HIV-1 Genotyping and Drug Resistance Mutations Mapping in Sicily, Italy

**DOI:** 10.3390/v17081129

**Published:** 2025-08-18

**Authors:** Luca Pipitò, Sara Cannella, Chiara Mascarella, Domenico Graceffa, Marcello Trizzino, Chiara Iaria, Pietro Colletti, Giovanni Mazzola, Giovanni M. Giammanco, Antonio Cascio, Celestino Bonura, Sicilian GRT Working Group

**Affiliations:** 1 Department of Health Promotion, Mother and Child Care, Internal Medicine, and Medical Specialties “G D’Alessandro”, University of Palermo, 90133 Palermo, Italy; sara.cannella@community.unipa.it (S.C.); chiara.mascarella@community.unipa.it (C.M.); giovanni.giammanco@unipa.it (G.M.G.); celestino.bonura@unipa.it (C.B.); 2 Infectious and Tropical Diseases Unit and Sicilian Regional Reference Center for the Fight Against AIDS, AOU Policlinico “P. Giaccone”, 90133 Palermo, Italy; marcello.trizzino@policlinico.pa.it; 3 Palermo Fast-Track City, Casa dei Diritti, 90100 Palermo, Italy; 4 Microbiology and Virology Unit, AOU Policlinico “P. Giaccone”, 90133 Palermo, Italy; domenico.graceffa@policlinico.pa.it; 5 Infectious Disease Unit, ARNAS Civico-Di Cristina, 90100 Palermo, Italy; iaria.chiara@gmail.com; 6 Unit of Infectious Diseaseas, Paolo Borsellino Hospital, ASP Trapani, 91025 Trapani, Italy; pietrocolletti1959@gmail.com; 7 Unit of Infectious Diseases, AOOR Villa Sofia-Cervello, 90100 Palermo, Italy; gnni.mazzola@gmail.com

**Keywords:** HIV, subtypes, GRT, genotypic resistance test, mutations, resistance, NGS, next generation sequencing

## Abstract

Background: The advent and continuous improvement in antiretroviral therapy (ART) have profoundly altered the clinical course of HIV infection, shifting the focus from AIDS-related complications to the management of age-related comorbidities and non-AIDS-related hospitalizations. In this evolving context, optimizing ART is essential, with genotypic resistance testing (GRT), particularly through next-generation sequencing (NGS), playing a pivotal role. Methods: This multicenter, retrospective cross-sectional study investigated HIV-1 subtypes, resistance mutations, and drug resistance profiles among 367 people living with HIV (PLWH) in Sicily, based on 384 GRTs performed at the Microbiology Laboratory of the University Hospital of Palermo. Results: Subtype B was the most prevalent (50%), followed by circulating recombinant forms (30%). Among treatment-naïve individuals, resistance-associated mutations were infrequent, with prevalence rates of 0.4% for NRTIs, 5.5% for NNRTIs, 1.3% for PIs, and 0.8% for INIs. Conversely, treatment-experienced individuals showed significantly higher resistance rates, especially to NRTIs (16.3%), NNRTIs (10.6%), and INIs (9.6%). No significant differences in resistance patterns were observed between B and non-B subtypes. Conclusions: This study provides the first regional overview of HIV drug resistance across Sicily. Despite the detection of resistance-associated mutations, the overall prevalence of clinically relevant resistance, particularly to currently recommended therapies, remains low, especially among treatment-naïve individuals.

## 1. Introduction

In the early years of the HIV epidemic, most patients succumbed to AIDS-related complications. However, the advent and progressive refinement of antiretroviral therapy (ART) have dramatically changed the natural history of HIV infection. In Italy, HIV testing, counseling, and ART are offered free of charge to all individuals regardless of nationality or immigration status. Testing is routinely available through public health services, hospital facilities, and community-based programs run by non-governmental organizations. Today, people living with HIV (PLWH) enjoy significantly longer life expectancy, shifting the clinical landscape toward the management of age-related comorbidities and non-AIDS-related hospitalizations [1,2,3]. In this evolving scenario, optimizing antiretroviral therapy is a key clinical goal. Genotypic resistance testing (GRT) plays a crucial role in this process, both in treatment-naïve individuals, where it provides a valuable baseline resistance profile that guides therapeutic decisions, and in patients experiencing virological failure, where the detection of resistance-associated mutations is essential for tailoring effective salvage regimens [4]. For clinicians and virologists alike, understanding the resistance landscape is crucial to ensure durable viral suppression and prevent the emergence of further mutations. Currently, next-generation sequencing (NGS) is the primary method used for GRT in Italy [5]. Compared to the previously used Sanger sequencing, NGS offers higher sensitivity, enabling the detection of low-abundance mutations within minor viral populations. This capacity allows clinicians to look beyond the “tip of the iceberg” and uncover hidden variants that may influence treatment response [6,7,8]. However, deeper sequencing is not always clinically advantageous. In some cases, the identification of mutations within non-replicating or non-competent viral populations may generate confusion rather than provide helpful guidance. For this reason, it is now widely accepted that only mutations present at a threshold of ≥10% or ≥20% should be considered clinically relevant and thus taken into account for therapeutic decisions [6,7,8]. Despite the increasing use of NGS for resistance testing, real-world data on the prevalence and clinical significance of mutations detected by this method remain limited, especially in some geographical areas. In Sicily, a region characterized by a diverse and heterogeneous population living with HIV, only one prior study has investigated HIV drug resistance using GRT, and it was limited to a single clinical center in Palermo [9]. The present study aims to fill this gap by analyzing the distribution of HIV-1 subtypes, mutations, and drug resistance profiles among PLWH attending multiple healthcare centers across Sicily, providing a regional overview of resistance patterns and viral diversity in this unique epidemiological setting. All genotypic analyses were centralized at the microbiology laboratory of the University Hospital in Palermo.

## 2. Materials and Methods

A cross-sectional study was conducted, including all PLHIV who underwent GRT at the Microbiology Laboratory of the University Hospital “Paolo Giaccone” in Palermo (Italy) between January 2022 and December 2024. Microbiological samples for GRT were submitted by Sicilian hospitals involved in HIV care. PLHIV were categorized as either treatment-naïve or treatment-experienced, who had experienced virological failure. Virological failure was defined as an HIV-1 RNA level above 200 copies/mL in PLHIV that initially achieved viral suppression (HIV-1 RNA levels below 50 copies/mL). In our setting, GRT is routinely performed at diagnosis in newly identified individuals and in patients experiencing virological failure, but not in those with sustained virological suppression. Therefore, the proportion of treatment-naïve individuals in our cohort does not reflect the overall proportion of treatment-naïve PLWH in Sicily, but rather the inclusion criteria of our study. Data on sex, age, and country of origin were collected retrospectively for each group and recorded in an anonymized database. HIV viral load and CD4 T cell count were collected for the naïve group, while ART history was collected for the experienced group. No data were available on transmission risk factors. GRT was performed for the protease, reverse transcriptase, and integrase genes for routine clinical purposes using viral RNA extracted from plasma samples. The commercial kit AD4SEQ HIV-1 Solution v2 (Arrow Diagnostics S.r.l., Genova, Italy) was used with the MiSeq Illumina platform for NGS analysis. Following sequencing, raw FastQ files were analyzed using the standalone software SmartVir (SmartSeq s.r.l., Alessandria, Italy), included with the commercial kit. The software queries the freely available web-based tool HIVdb ver 9.5.1 [http://hivdb.stanford.edu/ accessed 29 June 2025] to generate a report detailing all identified mutations and associated drug resistance. HIV-1 subtype determination was assessed using the same SmartVir software, which relies on the HIVdb version 9.5.1 algorithm for classification. Initially, the overall prevalence of mutations (major, minor, and accessory) was reported, including mutations not associated with drug resistance. Subsequently, antiretroviral drug susceptibility and resistance profiles were evaluated according to the HIVdb program integrated into the HIV-1 drug resistance database v.9.5.1. Resistance (R), reduced susceptibility (I), and susceptibility (S) were defined based on 10% and 20% NGS frequency cut-offs. Drug resistance results were visualized using bar charts. Treatment naïve and experienced individuals and B and non-B HIV-1 subtypes were compared for the prevalence of mutations and drug resistance. Statistical analyses were performed using the SPSS v.26.0 software package for Windows (IBM SPSS Statistics, ver. 26.0). Continuous variables were summarized as median and interquartile ranges, while categorical variables were presented as absolute and relative frequencies. The χ2 test or Fisher’s exact test was applied for comparison of categorical variables as appropriate.

## 3. Results

### 3.1. Features of PLWH Performing GRT

**Figure 1:**
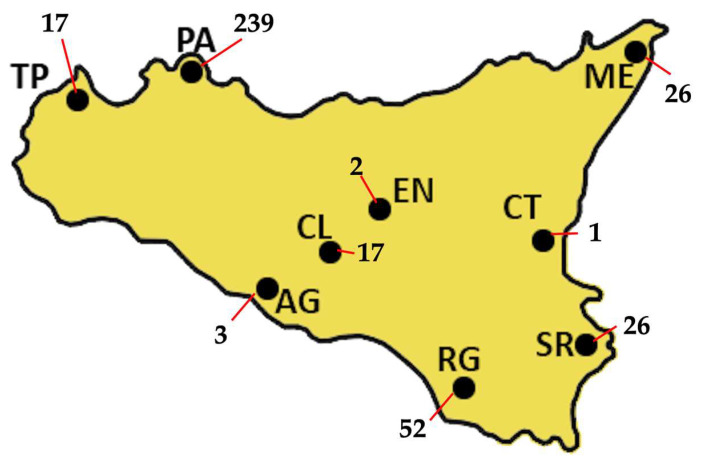
The absolute distribution of GRTs across Sicily, for a total of 383 GRTs, is shown. Most of the samples were collected in Palermo (PA), which alone accounts for 239 GRTs, representing 62.4% of the total. Ragusa (RG) follows with 52 GRTs (13.6%), while both Messina (ME) and Siracusa (SR) report 26 GRTs each, corresponding to 6.8% of the total. Trapani (TP) and Caltanissetta (CL) each produced 17 GRTs (4.4%), whereas Agrigento (AG) produced 3 (0.8%). Enna (EN) and Catania (CT) show the lowest frequencies, with only 2 GRTs (0.5%) and 1 GRT (0.3%), respectively.

A total of 384 GRTs were performed on 367 PLWH, including 238 treatment-naïve individuals, 89 treatment-experienced individuals, and 40 with unknown treatment status. Nine treatment-naïve PLWH repeated GRT because of treatment failure, while five treatment-experienced individuals had multiple GRTs. Figure 1 shows the absolute number of GRTs by centre, with 62.4% of GRTs coming from Palermo hospitals. The median age of the overall cohort was 44 years (IQR: 34–53). Among treatment-naïve individuals, the median age was 42 years (IQR: 33–52), with a male-to-female ratio of 1.6:1 (176 males to 38 females). Treatment-experienced patients had a median age of 48 years (IQR: 40–57), comprising 55 males and 32 females. In the naïve group, median HIV-1 viral load (VL) was 108,500 copies/mL (IQR: 16,325–525,000), and median CD4+ T-cell count was 188 cells/mm^3^ (IQR: 47–407). Among the experienced patients, prior to GRT, the most recent antiretroviral regimens included integrase strand transfer inhibitor (INI)-based therapies in 33 cases, protease inhibitor (PI)-based regimens in 14, non-nucleoside reverse transcriptase inhibitor (NNRTI)-based regimens in 6, and combined regimens in 18 patients.

### 3.2. HIV Subtypes

**Figure 2:**
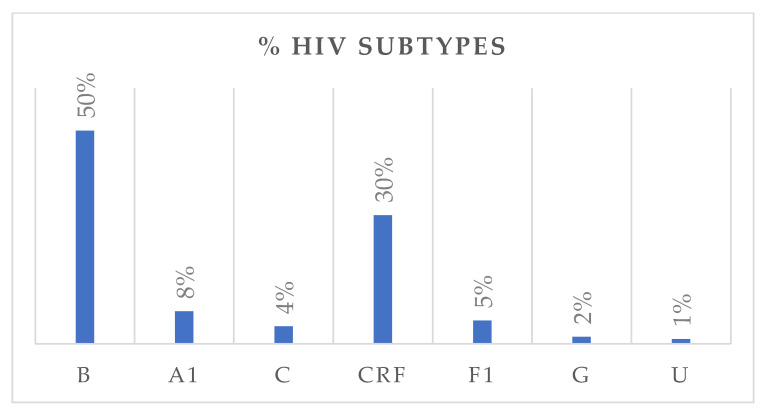
The bar chart illustrates the distribution of HIV-1 subtypes in the study population, expressed as percentages. U: unknown.

**Figure 3:**
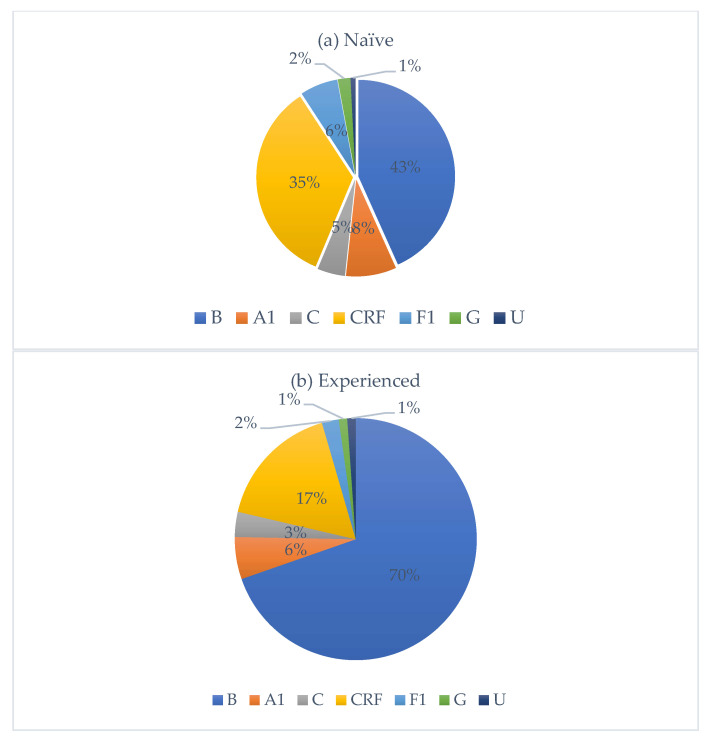
Distribution of HIV-1 subtypes among treatment-naïve (**a**) and treatment-experienced (**b**) individuals, respectively.

HIV subtype distribution is presented in Figure 2. Subtype B was the most prevalent, accounting for 50% of all cases, followed by circulating recombinant forms (CRFs) with 30%. Subtypes A1, F1, C, and G accounted for 8%, 5%, 4%, and 2%, respectively. Among CRFs, eleven subtypes were identified, with CRF02_AG being the most frequent (80%). Nationality data were available for 322 individuals. Of these, 251 were Italian, with the following HIV-subtype distribution: B (n = 161, M/F = 4.0), A1 (n = 13), C (n = 6), circulating recombinant forms (CRFs) (n = 53, M/F = 2.2), F1 (n = 14), and G (n = 3). Fifty-five individuals originated from African countries, with subtypes distributed as follows: B (n = 3), A1 (n = 3), C (n = 5), CRFs (n = 40, M/F = 1.4), and G (n = 2). An additional 16 individuals came from various other countries, showing a heterogeneous subtype distribution. Among treatment-naïve individuals, 67.1% were Italians, while 18.3% were non-Italians. Among treatment-experienced individuals, 73.9% were Italians, while 26.1% were non-Italians. Figure 3 depicts the distribution of HIV-1 subtypes according to treatment status. In the naïve group, subtype B was the most commonly observed (43%), followed by CRFs (35%), A1 (8%), C (5%), and F1 (6%). In contrast, the treatment-experienced group (Figure 3b) showed a markedly higher prevalence of subtype B (70%), while CRFs accounted for 17%, followed by A1 (6%), C (3%), and F1 (2%). Subtype G was rare in both groups.

### 3.3. Mutations and Drug Resistance

**Figure 4:**
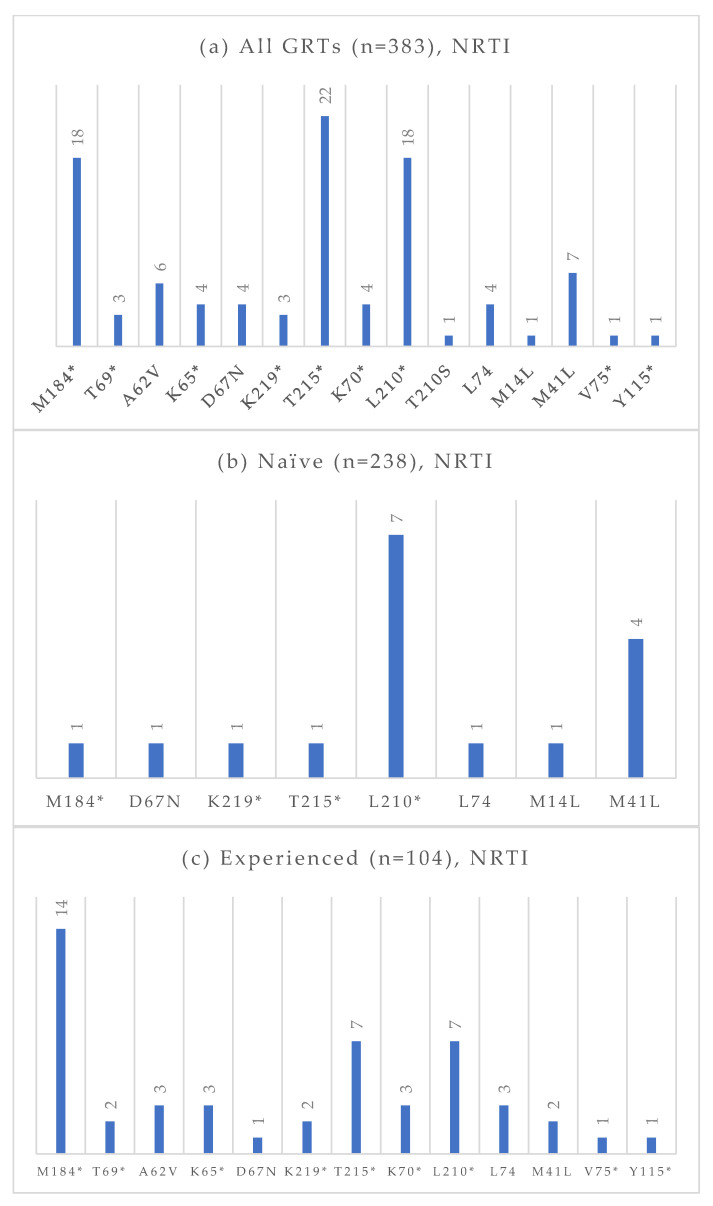
Absolute frequency of NRTI mutations according to treatment status: overall (**a**), treatment-naïve (**b**), treatment-experienced (**c**). Treatment-naïve: M184 (0.4%), D67N (0.4%), K219 (0.4%), T215 (0.4%), L210 (2.9%), L74 (0.4%), M14L (0.4%), M41L (1.7%). Treatment-experienced: M184 (13.5%), T69 (1.9%), A62V (2.9%), K65 (2.9%), D67N (0.9%), K219 (1.9%), T215 (6.7%), K70 (2.9%), L210 (6.7%), L74 (2.9%), M41L (1.9%), V75 (0.9%), Y115 (0.9%). * Notation used to indicate any amino acid substitution.

**Figure 5:**
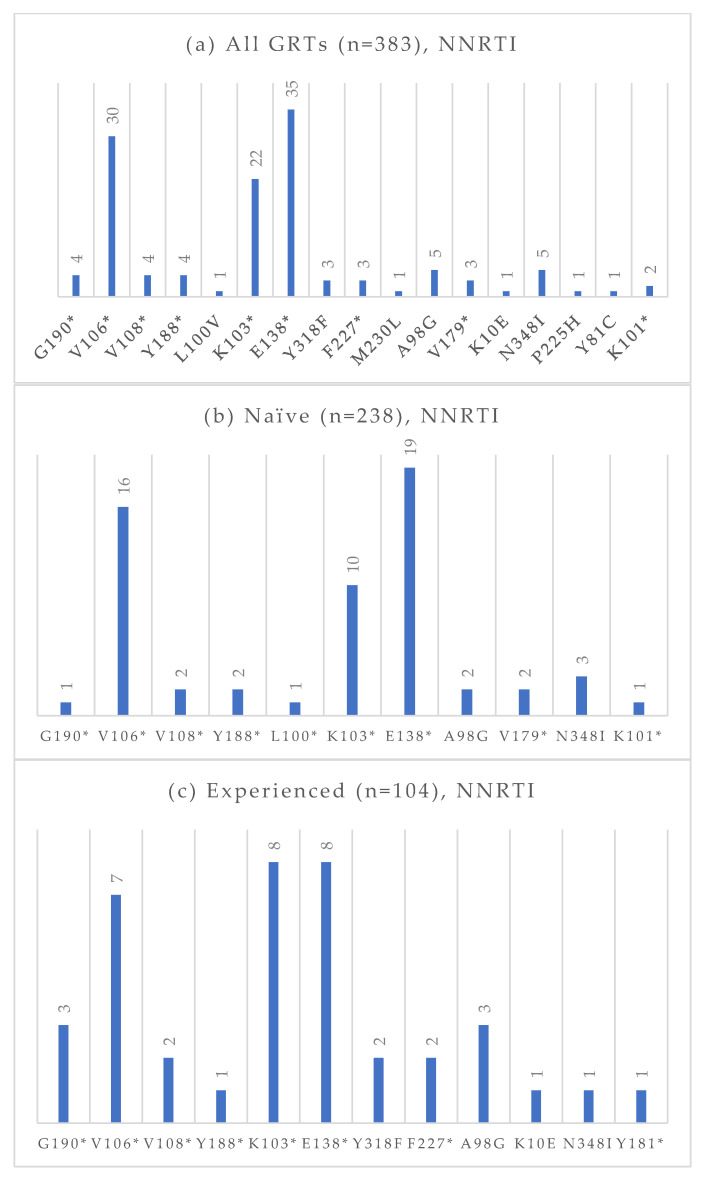
Absolute frequency of NNRTI mutations according to treatment status: overall (**a**), treatment-naïve (**b**), treatment-experienced (**c**). Treatment-naïve: G190 (0.4%), V106 (6.7%), V108 (0.8%), Y188 (0.8%), L100 (0.4%), K103 (4.2%), E138 (8.0%), A98G (0.8%), V179 (0.8%), N348I (1.3%), K101 (0.4%). Treatment-experienced: G190 (2.9%), V106 (6.7%), V108 (1.9%), Y188 (1.0%), K103 (7.7%), E138 (7.7%), Y318 (1.9%), F227 (1.9%), A98G (2.9%) K10E (1.0%), N348I (1.0%), Y181 (1.0%). * Notation used to indicate any amino acid substitution.

**Figure 6:**
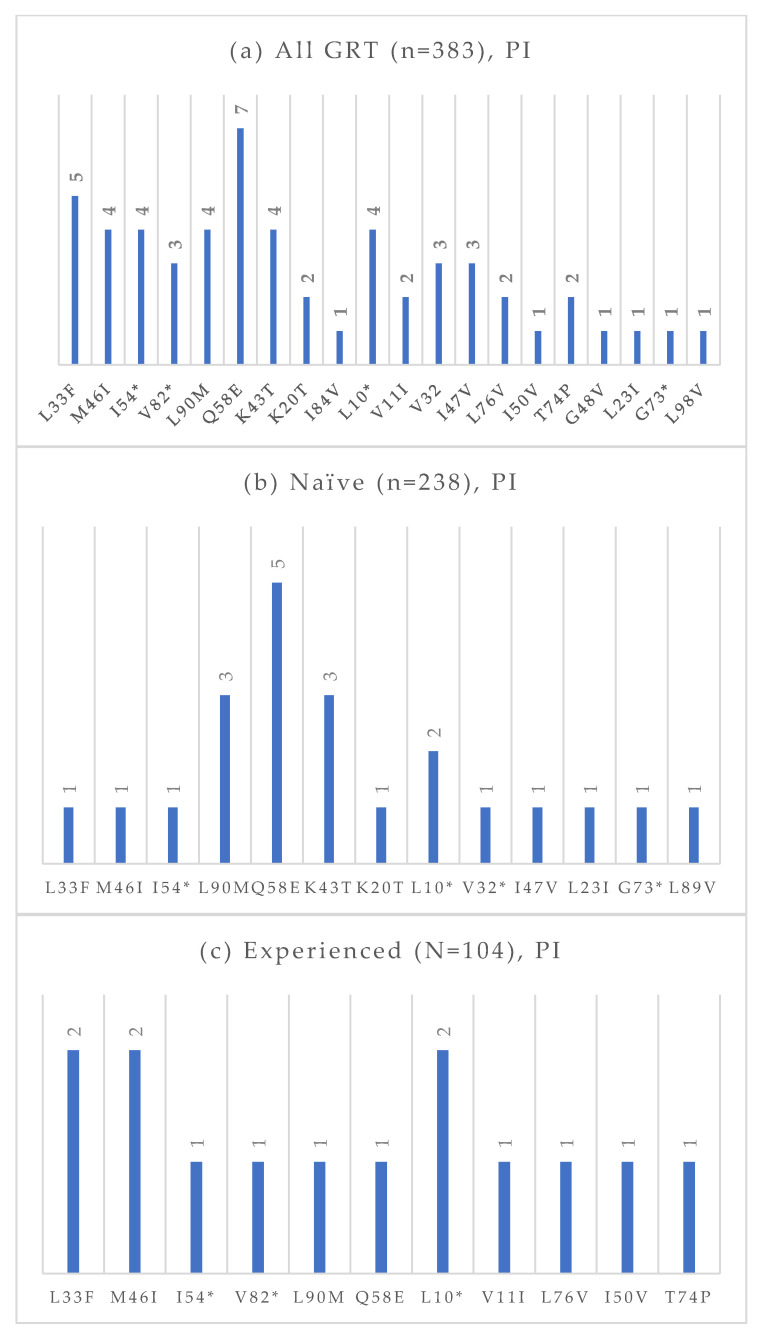
Absolute frequency of PI mutations according to treatment status: overall (**a**), treatment-naïve (**b**), treatment-experienced (**c**). Treatment-naïve: L33F (0.4%), M46I (0.4%), I54 (0.4%), L90M (1.3%), Q58E (2.1%), K43T (1.3%), K20T (0.4%), L10 (0.8%), V32 (0.4%), I47V (0.4%), L32I (0.4%), G73 (0.4%), L89V (0.4%). Treatment-experienced: L33F (1.9%), M46I (1.9%), I54 (1.0%), V82 (1.0%), L90M (1.0%), Q58E (1.0%), L10 (1.9%), V11 (1.0%), L76V (1.0%), I50V (1.0%), T74P (1.0%). * Notation used to indicate any amino acid substitution.

**Figure 7:**
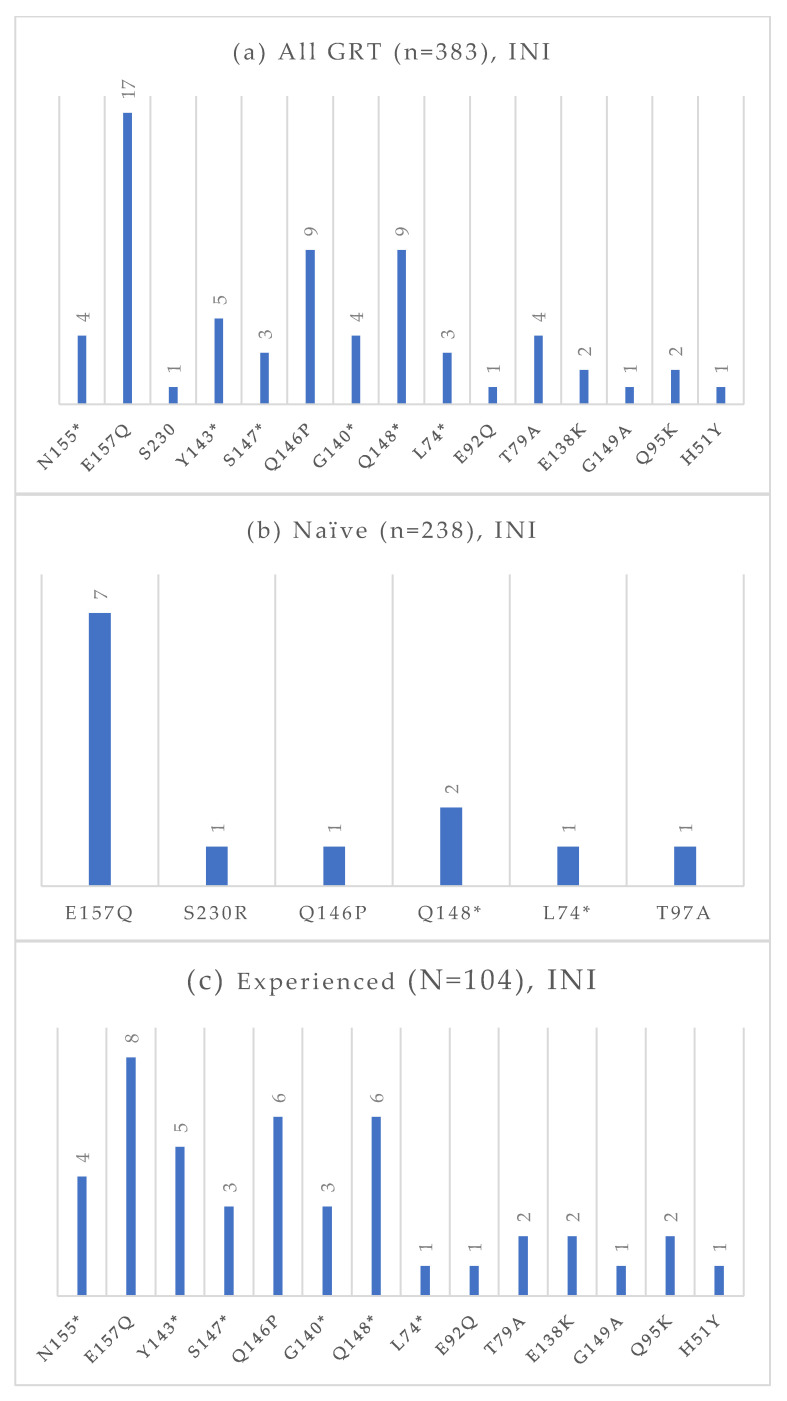
Absolute frequency of INI mutations according to treatment status: overall (**a**), treatment-naïve (**b**), treatment-experienced (**c**). Treatment-naïve: E157Q (2.9%), S30R (0.4%), Q146P (0.4%), Q148 (0.8%), L74 (0.4%), T97A (0.4%). Treatment-experienced: N155 (3.8%), E157Q (7.7%), Y143 (4.8%), S147 (2.9%), Q146P (5.8%), G140 (2.9%), Q148 (5.8%), L74 (1.0%), E92Q (1.0%), T97A (1.9%), E138K (1.9%), G149A (1.0%), Q95K (1.9%), H51Y (1.0%). * Notation used to indicate any amino acid substitution.

Among the 383 GRTs performed, 238 were conducted on treatment-naïve individuals, and 104 were conducted on treatment-experienced patients. The absolute frequencies of detected mutations, stratified by treatment status and antiretroviral drug class, are presented in Figure 4, Figure 5, Figure 6 and Figure 7. At an NGS threshold of 20%, the most frequent mutations identified in the treatment-naïve group were L210W (2.9%) and M41L (1.7%) among those NRTIs-associated, E138 (8.0%), V106 (6.7%) and K103 (4.2%) among NNRTIs, Q58E (2.1%) in the PI class, and E157Q (2.9%) in the INI class. In the treatment-experienced group, the most frequent mutations were M184 (13.5%) among NRTIs, E138 (7.7%), K103 (7.7%), V106 (6.7%) among NNRTIs, and E157Q (7.6%), Q148 (5.8%), and Q146P (5.8%) in the INI class. As in the naïve group, mutations in the PI class remained the least frequent in treatment-experienced individuals. When applying a lower NGS cut-off of 10%, only minor changes were observed in the prevalence of resistance-associated mutations. For NRTIs, one additional L210 mutation was detected in a treatment-naïve patient. For NNRTIs, six additional V106 mutations (three in treatment-naïve and one in treatment-experienced individuals) and one V179 mutation (in treatment-experienced individuals) were identified. For PIs, two I54 mutations (one treatment-naïve, one treatment-experienced), one V82 mutation (treatment-experienced), and one K43T mutation (treatment-naïve) were observed in addition. Among INI-associated mutations, additional findings included: one N155 mutation (unknown treatment status), one E157Q (treatment-experienced), two Y143 mutations (treatment-naïve), three S147 mutations (one treatment-naïve and two treatment-experienced), one G149A (unknown treatment status), and one H51Y (treatment-experienced). Considering PLWH previously exposed to INIs (n = 48), the most common INI-associated mutations were E157Q (n = 5, 10.4%), N155 (n = 4, 8.3%), Q146P (n = 4, 8.3%), and Q148 (n = 4, 8.3%).

In Table 1, mutation prevalences are summarized according to treatment status.

Resistance to at least one drug within each antiretroviral class was evaluated across the entire cohort of 383 individuals. The overall prevalence was as follows: NRTI resistance in 23/383 (6.0%), NNRTI resistance in 31/383 (8.1%), PI resistance in 7/383 (1.8%), and INI resistance in 13/383 (3.4%). Among PWH harboring INI-associated mutations (n = 33, 8.6%), 39.4% had at least one major mutation, whereas 97.0% had accessory mutations. In the treatment-naïve group, resistance was detected at substantially lower rates: NRTI resistance in 1/238 (0.4%), NNRTI in 13/238 (5.5%), PI in 3/238 (1.3%), and INI in 2/238 (0.8%). In contrast, treatment-experienced individuals showed higher resistance levels: NRTI in 17/104 (16.3%), NNRTI in 11/104 (10.6%), PI in 2/104 (1.9%), and INI in 10/104 (9.6%). For the INI class, among individuals exposed to integrase inhibitors, 8 (16.7%) displayed resistance. Of these, resistance was observed in all cases to raltegravir, in 7 to elvitegravir, in 4 to cabotegravir, in 2 to dolutegravir, and in 2 to bictegravir. Reduced susceptibility to cabotegravir, dolutegravir, and bictegravir was observed in 2, 3, and 3, respectively.

**Figure 8:**
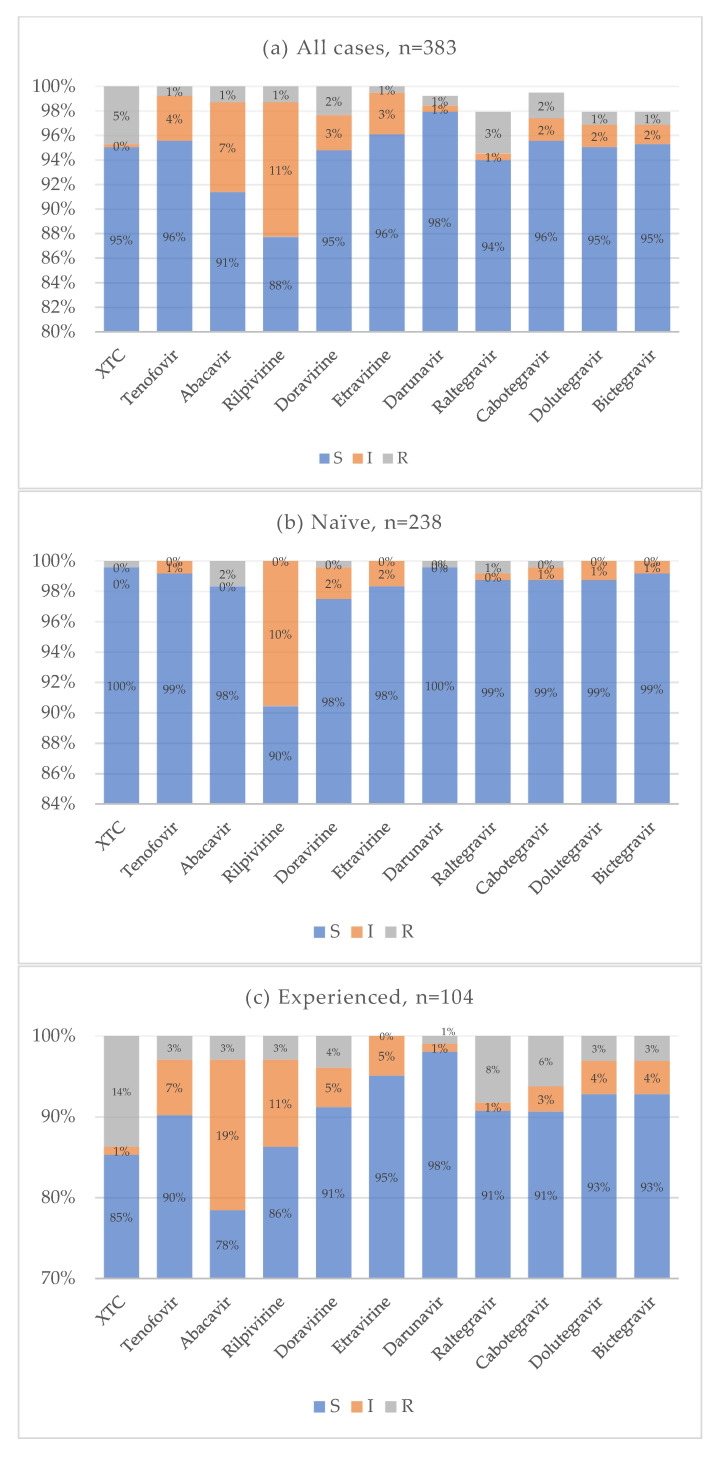
Cumulative antiretroviral resistance for all cases (**a**), naive (**b**), and experienced (**c**).

**Figure 9:**
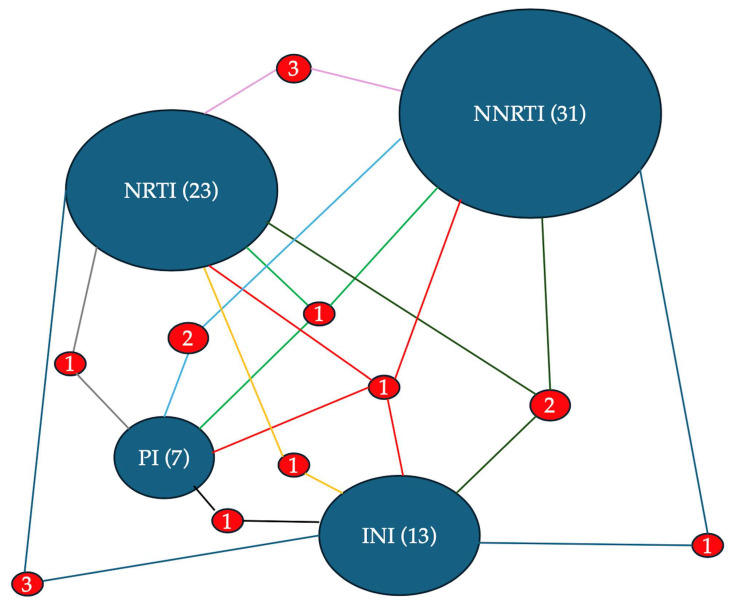
The diagram presents a network visualization of HIV drug resistance according to antiretroviral drug classes. The size of the ovals with the name of the drug class is proportional to the total number of associated resistances: NNRTI (31), NRTI (23), INI (13), and PI (7). The frequency with which mutations for the different classes are associated with each other is indicated by the number placed between the segments that join the two ovals. The size of the numbers and of the segment is proportional to the frequency of the association.

Cumulative resistance patterns for antiretroviral drug classes currently used in clinical practice are shown in Figure 8. In the treatment-naïve group, the overall prevalence of drug resistance was very low across all antiretroviral agents. Notably, 10% of naïve individuals exhibited reduced susceptibility to rilpivirine, while resistance to both first-generation and new-generation INIs remained low, around 1%. Conversely, treatment-experienced individuals showed a higher prevalence of resistance, particularly to emtricitabine and lamivudine. Resistance to INIs ranged from 8% for raltegravir to 3% for bictegravir and dolutegravir. The distribution of resistance to multiple drug classes is illustrated in Figure 9. In most cases, resistance was limited to one or two drug classes. Only a single individual showed resistance to at least one drug in each of the four antiretroviral classes. Statistical analysis showed that the treatment-naïve group had significantly lower resistance rates for NRTI (*p* < 0.001, OR = 0.021, CI: 0.003–0.160) and INI (*p* < 0.001, OR = 0.077, CI: 0.017–0.360) classes. No statistically significant differences were observed for NNRTI (*p* = 0.075) and PI (0.616) resistance. When analyzing specific mutations, the following were significantly less prevalent in treatment-naïve individuals: M184 (*p* < 0.001, OR = 0.025, CI: 0.003–0.192), E157Q (*p* = 0.020, OR = 0.307, CI: 0.108–0.873), Y143 (*p* = 0.028, OR = 0.142, CI: 0.027–0.748), S147 (*p* = 0.002, OR = 0.071, CI: 0.008–0.616), Q146P (*p* = 0.008, OR = 0.307, CI: 0.108–0.873), and Q148 (*p* = 0.008, OR = 0.142, CI: 0.027–0.748). Some mutations were reported only for the treatment-experienced group: A62V (*p* = 0.020, OR = 0), N155 (*p* = 0.005), G140 (*p* = 0.020). No significant differences for antiretroviral resistance or mutation prevalence were observed between subtype B and non-B HIV-1 subtypes.

## 4. Discussion

The use of NGS for GRT has become the gold standard for detecting HIV-1 mutations and resistance-associated variants. Despite being one of Italy’s largest regions, Sicily has been underrepresented in studies on this topic, with previous data limited to a single center in Palermo [9]. Our study expands the current understanding of HIV genetic diversity and resistance patterns in the region by analyzing a broader and more representative cohort. Our findings confirm the predominance of HIV-1 subtype B among Italian PLWH, followed by a substantial presence of CRFs, observed in both among Italian and non-Italian individuals. This reflects HIV-1 increasing genetic heterogeneity, consistent with global trends driven by migration and intercontinental transmission networks. In Sicily, CRF02_AG accounts for 80% of CRF cases and is more frequently observed among individuals originating from African countries. This distribution is consistent with known migration patterns from West and Central Africa. The male-to-female ratio was lower among individuals with CRF subtypes, particularly among African PLWH. These findings suggest that women infected with CRF02_AG who have recently immigrated may represent a key subgroup for targeted prevention and linkage-to-care strategies. Although our dataset did not include detailed migration histories, this epidemiological pattern underscores the importance of integrating molecular surveillance with socio-demographic data. Another notable difference in the male-to-female ratio was observed with respect to treatment status: among treatment-experienced patients, the proportion of females was higher than in the treatment-naïve group. A plausible explanation is that a substantial proportion of treatment-experienced individuals undergoing GRT were foreign-born, a population in which women are overrepresented and in which the pattern of risk factors differs from that currently observed in industrialized countries such as Italy. GRTs were analyzed using two NGS detection thresholds: 10% and 20%. The high concordance observed between the two thresholds suggests that a 20% cut-off remains reliable for routine resistance detection, with the 10% threshold providing only marginal additional sensitivity, though potentially relevant in selected clinical scenarios. European data show a declining trend in both transmitted and acquired HIV drug resistance from 1981 to 2019, with the most commonly detected mutations including K103, T215, M184, M41I/L, M46I/L, and L90M [10]. A similar decline has been observed in Italy, likely due to the higher genetic barrier and increased potency of newer antiretroviral agents [11]. Globally, the estimated prevalence of pretreatment transmitted resistance to any NRTIs (4%) and NNRTIs (6%) exceeds the rates observed in our cohort, being 0.4% for NRTIs and 5.5% for NNRTIs [12]. Similarly, global estimates of acquired resistance (58% for NRTIs and 67% for NNRTIs among treatment-experienced PLWH) were substantially higher than those observed in our study (16.3% for NRTIs and 10.6% for NNRTIs) [12]. When evaluating resistance to at least one drug within each antiretroviral class, we found notable prevalence rates: 6.0% for NRTIs, 8.1% for NNRTIs, 1.8% for PIs, and 3.4% for INIs. As expected, resistance was significantly more common among treatment-experienced individuals. In the treatment-naïve group, resistance to NRTIs and INIs was rare (0.4% and 0.8%, respectively), but much higher in experienced individuals (16.3% and 9.6%, respectively). Previous Italian studies using Sanger sequencing for detection of HIV mutations and resistance in treatment-naïve PLWH reported prevalence rates ranging from 1.3% [13] to 4.8% [14] for NRTIs, 3.4% [15] to 5.8% [16] for NNRTIs, 1% [16] to 3.9% [17] for PIs, and 0.3% [18] to 3.8% [16] for INIs. In contrast, higher resistance rates have been documented in treatment-experienced individuals [19,20,21,22], reaching as high as 82.4% for INIs in highly treatment-experienced cases [21].

In our study, NNRTI mutations were the most frequent among treatment-naïve individuals, particularly E138, V106, and K103. NRTI mutations such as L210W and M41L were uncommon, and PI and INI mutations were rare, with Q58E and E157Q being the most prevalent in their respective classes. Conversely, treatment-experienced individuals exhibited a higher overall prevalence of resistance-associated mutations, particularly in the NRTI and INI classes. The M184 mutation, linked to resistance to lamivudine and emtricitabine, was particularly frequent, likely reflecting prior exposure to these drugs. NNRTI mutations (E138, K103, and V106) were also commonly observed. INI resistance mutations were significantly more common among treatment-experienced patients, likely due to selective pressure from prior INI-containing regimens, which were frequently used in this group. Notably, only 3% of GRTs in experienced individuals showed resistance to dolutegravir and bictegravir.

Previous in vitro studies have demonstrated the high genetic barrier of second-generation INIs, while also highlighting the comparatively lower potency of cabotegravir relative to bictegravir and dolutegravir. Increasing concentrations of cabotegravir have been associated with the emergence of the Q148 mutation, which confers cross-resistance to all currently available INIs [23]. Among individuals experiencing virologic failure on cabotegravir, the most frequently selected mutations included Q148R, N155H, and E138K. In our cohort, overall resistance to cabotegravir was 2%, with Q148 mutations detected in nine cases, predominantly among treatment-experienced individuals. The low prevalence of resistance to both cabotegravir and rilpivirine among treatment-experienced patients supports the potential of long-acting therapies for individuals with suboptimal adherence to oral regimens. Second-generation INIs, particularly dolutegravir and bictegravir, retained activity against single major INI mutations, including Q148. However, their antiviral efficacy was significantly reduced when Q148 occurred in combination with two or more additional resistance-associated mutations [24]. Data on French and Italian patients who experienced virologic failure on dolutegravir identified N155H (5.4%), G140S (4.5%), Q148H (4.3%), and G138K (2.8%) as the most common mutations, with INI resistance primarily associated with prior exposure to first-generation INIs [25]. Between 2008 and 2017, a large Italian cohort of PLWH with prior INI exposure, most of whom experienced failure on INI-containing regimens, demonstrated low-level resistance to at least one INI in 42.9% of cases. The most frequently detected mutations were N155H, Q148, G140, E138, and Y143. Intermediate-level resistance to dolutegravir was observed in 15% of cases [20]. In our dataset, we observed a higher prevalence of N155H and Q148 mutations (8.3%) compared with the French and Italian cohorts. Considering the overall population, E157Q (7.6%) and Q146P (5.8%) emerged as the most prevalent INI mutations in our cohort. The E157Q polymorphism is considered an accessory INI resistance-associated mutation which, on its own, does not reduce susceptibility to either first- or second-generation INIs. However, it may contribute to resistance when present in combination with other mutations [26]. A further study reported E157Q in 4.1% of treatment-naïve individuals, without association with virologic failure on second-generation INIs [27]. Data on resistance-associated mutations to INIs remain limited. A study compared the prevalence of INI resistance in individuals who had never been exposed to INI-based regimens with that in INI-experienced patients, reporting overall low levels of resistance in both naïve and experienced groups. However, the latter group showed a higher prevalence of polymorphic, non-polymorphic, and rare mutations, with H155, S140, and H148 being the most frequently reported [28]. A multicenter European study found no signature INI-resistant variants circulating in Europe prior to the introduction of INIs; however, polymorphisms contributing to resistance were not uncommon [29]. A recent large-scale global analysis of sequences collected between 1983 and 2023 found that major INI resistance-associated mutations were rare in treatment-naïve individuals prior to the rollout of INIs [30]. Nevertheless, certain polymorphisms (e.g., E138K, G163K/R) were already present at low surveillance levels. The estimated global prevalence of major INI resistance-associated mutations over four decades was 0.35%. Paradoxically, a statistically significant decrease was observed in 2008–2023 compared with 1983–2007. This decline during the INI era is likely attributable to the availability of more effective ART regimens and improved adherence among treatment-experienced populations. In contrast, the prevalence of accessory INI resistance-associated mutations over the same forty-year period was 46.61%, with rates of 22.26% in 1983–2007 and 24.29% in 2008–2023 [30]. A Turkish study including 50 ART-naïve patients, 69 INI-free ART-experienced patients, and 82 INI-experienced patients detected no INI resistance in ART-naïve individuals, while prevalence rates were 10% in INI-free ART-experienced patients and 29% in INI-treated patients, predominantly involving first-generation INIs [31]. Similarly, a study conducted in New Orleans focusing exclusively on susceptibility to raltegravir and elvitegravir showed that INI resistance was associated with prior INI exposure; reduced susceptibility to either drug was observed in 14 of 41 (34%) INI-experienced patients with virological failure [32]. Loosli et al. reported that among individuals with viraemia while on dolutegravir-based ART, INI resistance-associated mutations and dolutegravir resistance were uncommon, with INI resistance-associated mutations detected in 14% (86/599) of cases and high-level dolutegravir resistance observed in only six individuals [33]. In clinical trials, the prevalence of virological failure with emergent INI resistance-associated mutations in PLWH receiving dolutegravir-containing regimens has been low, generally <0.1%, but higher among ART-experienced PLWH with virological suppression receiving dolutegravir monotherapy (3.4%) and among ART-experienced PLWH with virological failure on a previous regimen receiving dolutegravir plus two NRTIs (1.5%) [34]. More recently, Merad et al. demonstrated that archived INI resistance-associated mutations did not compromise virological control in PWH on suppressive INI-based therapy, as assessed through proviral genotypic resistance testing [35]. Collectively, these findings indicate that while the prevalence of INI resistance is higher among INI-exposed patients, it is predominantly associated with first-generation INIs. Resistance to second-generation INIs remains rare, consistent with the findings observed in our cohort. The PI class remained the least affected by resistance mutations, both in treatment-naïve and treatment-experienced individuals. Finally, our cumulative resistance analysis showed that the overall prevalence of resistance to currently used antiretroviral agents remained low, even among treatment-experienced individuals. In this group, resistance was most observed against emtricitabine and lamivudine, while resistance to INIs ranged from 8% for raltegravir to 3% for bictegravir and dolutegravir. Multi-class resistance was uncommon; most individuals exhibited resistance to only one or two drug classes. Only one patient exhibited resistance to at least one agent in each antiretroviral class, underscoring the rarity of pan-resistance in our setting. No significant differences in drug resistance prevalence or mutation profiles were observed between subtype B and non-B strains.

## 5. Conclusions

In conclusion, our findings highlight the utility of NGS for detailed HIV-1 resistance profiling, confirming the predominance of subtype B in Sicily, alongside the emergence of CRFs reflecting both regional and international transmission dynamics. Despite the presence of resistance-associated mutations, the overall prevalence of clinically relevant resistance, particularly to drugs currently used in first-line regimens, remains low, especially among treatment-naïve individuals. These results support the sustained efficacy of current therapeutic options and highlight the importance of ongoing molecular surveillance to inform regional HIV treatment strategies, especially for the management of treatment-experienced individuals.

## Figures and Tables

**Table 1:** Mutation prevalence according to treatment status.

Mutations	Overall (n = 383)	Naïve (n = 238)	Experienced (n = 104)
NRTI			
M184 *	18 (4.7%)	1 (0.4%)	14 (6.8%)
T69 *	3 (0.8%)	0	2 (1.9%)
A62V	6 (1.6%)	0	3 (2.9%)
K65 *	4 (1.0%)	0	3 (2.9%)
D67N	4 (1.0%)	1 (0.4%)	1 (1.0%)
K219 *	3 (0.8%)	1 (0.4%)	2 (1.9%)
T215 *	22 (5.7%)	1 (0.4%)	7 (6.7%)
K70 *	4 (1.0%)	0	3 (2.9%)
L210 *	18 (4.7%)	7 (2.9%)	7 (6.7%)
T210S	1 (0.3%)	0	3 (2.9%)
L74	4 (1.0%)	1 (0.4%)	3 (2.9%)
M14L	1 (0.3%)	1 (0.4%)	0
M41L	7 (1.8%)	4 (1.7%)	2 (1.9%)
V75 *	1 (0.3%)	0	1 (1.0%)
Y115 *	1 (0.3%)	0	1 (1.0%)
NNRTI			
G190 *	4 (1.0%)	1 (0.4%)	3 (2.9%)
V106 *	30 (7.8%)	16 (6.7%)	7 (6.7%)
V108 *	4 (1.0%)	2 (0.8%)	2 (1.9%)
Y188 *	4 (1.0%)	2 (0.8%)	1 (1.0%)
L100V	1 (0.3%)	1 (0.4%)	0
K103 *	22 (5.7%)	10 (4.2%)	8 (7.7%)
E138 *	35 (9.1%)	19 (8.0%)	8 (7.7%)
Y318F	3 (0.8%)	2 (0.8%)	2 (1.9%)
F227 *	3 (0.8%)	0	2 (1.9%)
M230L	1 (0.3%)	0	0
A98G	5 (1.3%)	2 (0.8%)	3 (2.9%)
V179 *	3 (0.8%)	2 (0.8%)	0
K10E	1 (0.3%)	0	1 (1.0%)
N348I	5 (1.3%)	3 (1.3%)	1 (1.0%)
P225H	1 (0.3%)	0	0
Y181	0	0	1 (1.0%)
K101 *	2 (0.5%)	(0.4%)	0
PI			
L33F	5 (1.3%)	1 (0.4%)	2 (1.9%)
M46I	4 (1.0%)	1 (0.4%)	2 (1.9%)
I54 *	4 (1.0%)	1 (0.4%)	1 (1.0%)
V82 *	3 (0.8%)	0	1 (1.0%)
L90M	4 (1.0%)	3 (1.3%)	1 (1.0%)
Q58E	7 (1.8%)	5 (2.1%)	1 (1.0%)
K43T	4 (1.0%)	3 (1.3%)	0
K20T	2 (0.5%)	1 (0.4%)	0
I84V	1 (0.3%)	0	0
L10 *	4 (1.0%)	2 (0.8%)	2 (1.9%)
V11I	2 (0.5%)	0	1 (1.0%)
V32	3 (0.8%)	1 (0.4%)	0
I47V	3 (0.8%)	1 (0.4%)	0
L76V	2 (0.5%)	0	1 (1.0%)
I50V	1 (0.3%)	0	1 (1.0%)
T74P	2 (0.5%)	0	1 (1.0%)
G48V	1 (0.3%)	0	0
L23I	1 (0.3%)	1 (0.4%)	0
G73 *	1 (0.3%)	1 (0.4%)	0
L90V	1 (0.3%)	1 (0.4%)	0
INI			
N155 *	4 (1.0%)	0	4 (3.8%)
E157Q	17 (4.4%)	7 (2.9%)	8 (7.7%)
S230R	1 (0.3%)	1 (0.4%)	0
Y143 *	5 (1.3%)	0	5 (4.8%)
S147 *	3 (0.8%)	0	3 (2.9%)
Q146P	9 (2.3%)	1 (0.4%)	6 (5.8%)
G140 *	4 (1.0%)	0	3 (2.9%)
Q148 *	9 (2.3%)	2 (0.8%)	6 (5.8%)
L74 *	3 (0.8%)	1 (0.4%)	1 (1.0%)
E92Q	1 (0.3%)	0	1 (1.0%)
T97A	4 (1.0%)	1 (0.4%)	2 (1.9%)
E138K	2 (0.5%)	0	2 (1.9%)
G149A	1 (0.3%)	0	1 (1.0%)
Q95K	2 (0.5%)	0	2 (1.9%)
H51Y	1 (0.3%)	0	1 (1.0%)

NRTI: nucleoside reverse transcriptase inhibitor; NNRTI: non-nucleoside reverse transcriptase inhibitor; PI: protease inhibitor; INI: integrase strand transfer inhibitor; * notation used to indicate any amino acid substitution.

## Data Availability

Data are available on request to the corresponding authors. All the sequences used in this study are available in the Italian ARCA database (https://www.dbarca.net), which can be accessed upon request to the corresponding author.

## Abbreviations

The following abbreviations are used in this manuscript:

ART	Antiretroviral therapy
CRF	Circulating recombinant forms
GRT	Genotyping resistance test
HIV	Human immunodeficiency virus
INI	Integrase strand transfer inhibitor
NGS	Next-generation sequencing
NRTI	Nucleoside reverse transcriptase inhibitor
NNRTI	Non-nucleoside reverse transcriptase inhibitor
OR	Odds ratio
PI	Protease inhibitor
PLWH	People living with HIV
